# Focal screening and treatment around passively-detected malaria cases in Pailin Province, Cambodia: a feasible and effective tool to target asymptomatic infections?

**DOI:** 10.1186/1475-2875-13-S1-P45

**Published:** 2014-09-22

**Authors:** John Hustedt, Sara Canavati, Chandary Rang, Sophal Uth, Po Ly, Siv Sovannaroth, Ruth Ashton, Sylvia Meek, Arantxa Roca Felter

**Affiliations:** 1 Malaria Consortium, Phnom Penh, Cambodia; 2 Malaria Consortium, Pailin, Cambodia; 3 National Centre for Parasitology, Entomology, and Malaria Control, Phnom Penh, Cambodia; 4 Malaria Consortium, London, UK; 5 Mahidol Oxford Tropical Medicine Research Unit, Phnom Penh, Cambodia

## Background

Pailin Province in Cambodia is a malaria pre-elimination setting, with very low levels of Plasmodium transmission. Malaria elimination in this location is a priority, due to the confirmed presence of artemisinin-resistant parasites. The objective of this study was to evaluate the feasibility of a focal screening and treatment (FSAT) approach using microscopy, rapid diagnostic tests (RDT) and polymerase chain reaction (PCR) around passively-detected Plasmodium infections, as well as to assess the impact of FSAT in identifying and eliminating the asymptomatic reservoirs of Plasmodium in the population.

## Materials and methods

Following passive identification of Plasmodium infected patients by health staff with RDT, targeted screening was conducted either i) among all additional residents of the index case household (nHH = 255); ii) all residents of the five households closest to the index household (nHH = 45); or iii) all residents of the closest 10 households (nHH = 80). Screening was conducted within three days of the index case being identified. In addition, 60 households within the health centre catchment population were randomly selected for screening. Half of these households were selected from villages with >10 confirmed malaria cases in 2013, and half from villages <10 cases. Individuals provided a blood sample onsite for microscopy, RDT, and PCR analysis. Plasmodium genotyping analysis was conducted to investigate whether index and non-index infections are linked.

## Results

A total of 270 index cases (symptomatic, RDT-positive) presented to health workers over the nine month study period, prompting screening activities in the community. Of a total 1869 non-index cases tested by PCR, 43 (2.3%) were positive for Plasmodium infection; 25 *P. falciparum*, 16 *P. vivax* and two mixed infections. We present these PCR data stratified by household selection criteria (index, nearby and randomly selected) to explore if or at what level FSAT around passively-detected symptomatic infections is useful in this context. These data are also presented for each diagnostic tool. Full details of risk factors for symptomatic and asymptomatic infection in this population will be presented. These data provide additional evidence to assess the usefulness of focal screening around symptomatic malaria cases as a strategy to identify further asymptomatic infections in the population. The potential effectiveness of this approach in a malaria elimination programme is discussed.

